# Meddlesome Instrumentation in Urethral Diseases*Read at the meeting of the Georgia Medical Association, Macon, April, 1897.

**Published:** 1897-06

**Authors:** W. L. Champion

**Affiliations:** Atlanta, Ga.


### MEDDLESOME INSTRUMENTATION IN URETHRAL DISEASES.*

By W. L. CHAMPION, M.D.,
ATLANTA, GA.

The ugly train of symptoms that follow in the wake of bad urethral surgery, and the results obtained from such instrumentation is frequently a monument as lasting to the surgeon as a fracture improperly treated. It seems the impression is prevalent in the minds of some, that the urethra has no function at all, but was made for the surgeon's use to demonstrate his skill in the use of instruments. Considering the teachings of to-day and our knowledge of the importance of cleanliness in surgery, it is a puzzle to know why physicians continue to thrust dirty instruments, made sleek with rancid grease, into the urethra and bladder, producing untoward results and not knowing the source of infection. If the instruments are clean, it frequently happens that they are passed into the bladder, carrying purulent material retained within the urethra. While a diseased urethra is as a rule an unclean canal, it frequently happens that the deep urethra and bladder are in a healthy state, so there is no common sense in "adding fuel to the fire" by using unclean instruments or forcing poisonous material into uninfected areas. The close observer rarely overlooks the origin of urethral fever, swelled testicle, cystitis, prostatitis, damaged kidneys and many serious conditions directly due to the meddlesome use of instruments. Not to be a meddler in the treatment of genito-urinary diseases, it is essential to be familiar with the use of instruments; to know when to use them, and what kind of instruments to use.

The use of small steel instruments, below 18 or 20 French cannot be too strongly condemned. With our knowledge of the anatomy of the urethra, and of the dangers of passing small steel instruments, false passages should be a thing of the past. The soft bougies, though not as durable as the steel sounds, accomplish the same results, and should always be used when a small instrument is called

* Read at the meeting of the Georgia Medical Association, Macon, April, 1897.

for; and even the larger ones are just as serviceable and produce less pain on introduction.

The routine practice of passing sounds *into the bladder* in treating strictures in the penile portion of the urethra, is not only useless but bad surgery. There is always a liability of infecting the bladder, and producing irritation of the prostatic urethra. The short, straight sound passed through the stricture accomplishes the same result as the curved instrument, and the danger of producing complications is lessened.

An important point that should never be overlooked, is the necessity of having instruments perfectly clean that are to be introduced into the bladders of old men with enlarged prostate; and this point should always be impressed upon the patient when he is given a catheter to use.

The carelessness with which instruments are thrust into the urethra frequently results in permanent injury to the tissues. "It is a very easy thing to force a catheter or sound through the urethral walls, or to produce sufficient injury by bruising and laceration to result in cicatricial deposit and consequent stricture." This is especially true when the canal is highly inflamed; and probably many of you have had cases of cystitis, and later organic stricture to treat due to meddlesome surgery of this kind.

The custom of many physicians of using the steel sound for exploring the urethra, to determine whether stricture is present or not, should be abandoned. The sound is practically worthless as an instrument to arrive at any knowledge as to the condition of the urethra. Patients frequently present themselves for treatment who have been examined with sounds and told there were no strictures present, when a proper examination would reveal a badly strictured canal. A stricture that can be detected by a 25 bulbous bougie will frequently admit a thirty sound, and for this reason errors in diagnosis are made.

To meet with success in the treatment of urethral diseases it is necessary to make a careful examination. Neglect in this particular is why many failures are recorded. Patients with contracted meatus that will admit only a 26 or 28 bulbous bougie (urethrameter not being used) are told that they have no stricture, when it is impossible to determine whether the urethra is free of strictures until a bulbous bougie as large as the urethra is introduced.

The mistaken idea that every apparently gleety discharge from the urethra is an indication for the use of the sound, is clearly shown in the discharge due to prostatic congestion, discharge from gonorrheal inflammation of the seminal vesicles, the discharge of long standing after epididymitis, the discharge we frequently see from syphilitic mucous patches within the urethra, and other conditions that respond to proper treatment.

When a patient presents himself for treatment, if he has a highly sensitive urethra or discharge from the canal, first treat the urethra by irrigation until the sensitive condition has disappeared and discharge has been controlled, before making an examination or commencing treatment with instruments.

In treating stricture of the urethra by dilatation how often should we pass an instrument? This question has been written upon, argued and discussed at length, and there seems to be a wide variance of opinion as to the length of time that should elapse between the sittings. In the use of sounds for the treatment of stricture there can be no fixed law in regard to the intervals to be allowed between the sittings. Each case must be watched separately, and the results of the introduction of the instrument noted, so as to determine when to use the instrument again. My opinion is that the majority of men not studying the effects produced by an instrument passed through an organic stricture, influenced by the patient's desire for a rapid cure, are prone to pass instruments too often, thereby setting up an acute inflammatory condition and prolonging the treatment.

My experience in the treatment of urethral strictures coincides with that of Dr. Keyes, of New York. Probably most of you know his views on this question, but I will quote him at length so as to be plainly understood. He says: "Suppose a stricture which sensibly diminishes the size of the stream of urine, and is attended by gleet. Through this stricture a conical instrument is introduced, which is arrested for a moment, but gradually passes, stretching the stricture, and is distinctly 'grasped' as it is being withdrawn. What follows such an operation? At the next act of urination the stream is larger, and continues so during twenty-four hours. At the end of that time the stream is nearly as small as it was before the sound was used; the gleet is the same, or possibly increased. Now, for twenty-four to forty-eight hours the stream steadily becomes smaller, while the discharge grows more abundant and creamy. During the third or fourth day, improvement commences; the stream again grows larger, the discharge becomes thinner and less copious, and this improvement often continues through the fifth and sixth or even seventh days, or longer, after which the volume of the stream commences to diminish and the discharge to become thicker. In such a case, if the same conical instrument first used had been reintroduced at the end of twenty-four hours, it would have passed the stricture with about the same facility as on the day before; if after forty-eight hours, it would enter with more difficulty; if at the end of seventy-two hours, it would again enter as easily as on the first day; if reintroduction were first attempted on the fourth day, the sound would pass more easily than at first; if on the fifth, with more ease still, and it would not probably be so tightly 'grasped' on withdrawal; while in some cases the greatest ease of reintroduction is attained on the sixth, seventh, eighth day, or even later. This varies in different cases; but it may be stated, as a rule, *that it is bad surgery, in treating stricture by dilatation, to reintroduce an instrument—unless it be filiform—before the lapse of at least seventy-two hours, and that more rapid progress will be made with the case by waiting till after ninety-six hours—often even until the sixth, seventh or eighth day.*"

If any one doubts the truthfulness of the above statement, all that is necessary to be convinced to the contrary is to watch the effect of an instrument under the same circumstances.

The cure of a stricture by dilatation is brought about by "absorption" of cicatricial tissue, so if the stricture is not stretched up to the full size of the urethra, the cure is not perfected, and recontraction will in all probability take place. This holds true in regard to strictures that have been cut; and a large percentage of the urethrotomies that are not successful are due to neglected after treatment. If a stricture can be cured by dilatation, the surgeon is not justifiable in subjecting the patient to a cutting operation, that might possibly prove fatal. Strictures of recent formation, situated in the pendulous portion of the urethra, can usually be cured by dilatation, and those in the deep urethra respond readily to this method.

Now, in regard to internal urethrotomy in the deep urethra, I know there are men who do this operation with but few accidents, but the best practice is to positively refuse to interfere unless the patient will submit to the external operation. The custom of doing internal urethrotomy in the office, and allowing the patient to go home in a hack or on a car cannot be too strongly condemned. It is an operation that is fraught with danger, so we should guard against any mishap, and give the patient the best treatment possible. The operation should be done at home, and the patient kept in bed for at least five days, and it would be safer still to require a week's rest.

The mere cutting of a stricture will never cure it; it is absolutely necessary to keep the urethra dilated up to its full size until perfect healing takes place, and all cicatricial tissue has disappeared as far as the sound will accomplish.

Before a stricture is cut the size of the urethra should be determined, and the stricture cut to this size. Then introduce a bulbous bougie and see if the slightest band of cicatricial tissue is left; if so reintroduce the urethratome and cut all remaining bands. If the word meddlesome applies to any operator, it is the one who cuts a stricture to number 32 when the caliber of the urethra is 34, and never introduces a sound larger than the size the stricture was cut to. An operation of this kind cannot give relief; for in the future there will probably be a recontraction of the stricture. The same kind of operative work is done in cutting the meatus: it is either not cut to the full size of the urethra, or neglected and not kept dilated until healing takes place.

It is not my intention to appear dogmatic in regard to these apparently simple operations, but I so frequently see strictures that were improperly treated, and meatus that have to be incised a second time, that I cannot refrain from emphasizing these important points.

In my opinion, after internal urethrotomy instruments are frequently passed too often; every fourth, fifth, or sixth day will give just as good results, and less pain to the patient, as when passed on the second and third. The nearer the stricture is to the meatus the shorter should be the intervals between the sittings.

The habit of passing sounds to cure a gleet, whether due to a stricture or not, is the cause of a twenty-five or twenty-six French, the largest the meatus will admit, to be thrust into the urethra to cure a chronic gonorrhea, when a mere incision of the meatus to its proper size, and local applications to the diseased surface, will perfect a cure.

It is bad practice to try and force an instrument through a stricture; it always results in damage to the urethra. If a stricture is of such small caliber that it seems impossible to pass an instrument at all, I feel sure that in many cases, if a stream of water is thrown against the stricture with an irrigator for a few minutes, that an instrument can be passed into the bladder. Under similar circumstances, if a two or four per cent. solution of cocaine is deposited at the stricture, it will produce contraction of the engorged vessels and tissues to such a degree that a filiform, or even larger instrument, can be passed.

The deep injection syringe, an instrument that is in general use, and one no doubt that has served a good purpose, is inferior to the endoscope. With the syringe it is a matter of guesswork whether the solution is deposited at the seat of inflammation; but with the endoscope we can make a careful examination of the entire urethra, and limit the application to the diseased surface.

Every discharge from the urethra of long standing is not due to a stricture that requires cutting or dilatation. In many cases the discharge is due to a chronic inflammation that produces a slight thickening of the urethra, and is diagnosed and occasionally operated upon as a stricture of large caliber. Strictures of this kind, if they can be called strictures, are the ones that give such quick and happy results from direct applications through the endoscope.

The granular condition that remains after a stricture has been dilatated or cut to its full size, will respond more promptly to local applications through the endoscope than other means. While the sound and deep injection syringe was originally and is yet used for the relief of these conditions, it is impossible to know the condition the urethra is left in when the patient is dismissed.

I frequently have patients who, having had a gleety discharge from the urethra for years, and having been treated with sounds for stricture until completely disgusted with instruments, make a permanent cure of the granular condition by a dozen or fifteen applications through the endoscope. The endoscope I use, and think one of the best, is Otis's instrument, with electric attachment, and Klotz tubes. The ordinary urethral tubes, with a head mirror to reflect the light, will not give satisfaction.

It is necessary to the surgeon's success and the patient's welfare to ascertain the condition of the patient's kidneys before doing an operation upon the urethra. I remember very well a case where an external urethrotomy was absolutely necessary to relieve a tight stricture of the deep urethra, that had caused a rupture, and the patient died from suppression of urine fifteen days after the operation, due to structural derangement of the kidney that was present before the operation. In urethral surgery preparatory treatment is very necessary. The bowels and kidneys should be in good working order, the urine should be rendered aseptic as far as possible, the parts to be operated upon should be thoroughly cleansed, and a dose of quinine before and after the operation is advisable. Taking these precautions will, I feel satisfied, reduce the mortality in surgical operations upon the urethra.

*In closing, I would like to emphasize the importance of irrigation before surgical operations upon the urethral canal, whether it be only the passage of a sound or an internal urethrotomy.* If this is done there will be fewer cases of urethral fever and less irritation and inflammation after using instruments. Within the past twelve months I have used "hydrostatic irrigation" in the treatment of inflammatory conditions of the urethra and bladder, and it is far superior to any other method. The container, which holds a prescribed quantity of the solution to be used, at a temperature of 110° F., is placed eight or nine feet from the floor. A glass nozzle is used to throw the fluid into the urethra and bladder. The anterior urethra is first thoroughly washed out, and then the nozzle is pressed firmly against the meatus, and the patient told to breathe deeply or try to urinate, and the fluid flows back into the bladder. When the bladder is full the patient is allowed to pass it out, and the bladder is refilled. The value of this method is that the urethra is distended to its full capacity, which forces the pus and germs from the glands and follicles of the canal.

*Bibliography.*—G. Frank Lydston, M.D., "On Stricture of the Urethra." E. L. Keyes, M.D., "Genito-Urinary Diseases and Syphilis." Prince A. Morrow, M.D., "A System of Genito-Urinary Diseases, Syphilology and Dermatology."

